# Aquatic exercises combined with cognitive tasks for older women (WaterCog Study): protocol for a randomized clinical trial

**DOI:** 10.1186/s12877-026-07084-8

**Published:** 2026-03-07

**Authors:** Cristine Lima Alberton, Mariana Borba Gomes, Henrique Santos Ferreira, Franciele Berní de Oliveira, Anna Ogonowska-Slodownik, Javier Güeita-Rodríguez, Thais Reichert, Maíra Junkes-Cunha, Victor Hugo Guesser Pinheiro, Luana Siqueira Andrade

**Affiliations:** 1 School of Physical Education and Physiotherapy, Federal University of Pelotas, Pelotas, Brazil; 2 Latinoamérica y Caribe (RIES-LAC), Red Interuniversitaria de Envejecimiento Saludable, Talca, Chile; 3 Faculty of Rehabilitation, Jozef Pilsudski University of Physical Education in Warsaw, Warsaw, Poland; 4 KITE Research Institute, Toronto Rehabilitation Institute, University Health Network, Toronto, Canada; 5 Physiotherapy, Occupational Therapy, Rehabilitation, and Physical Medicine Department/Health Sciences, Rey Juan Carlos University, Madrid, Spain; 6 Department of Physical Education, Federal University of Parana, Curitiba, Brazil; 7 Faculty of Health Sciences, University of Malaga, Malaga, Spain

**Keywords:** Aging, Physical exercise, Physical activity, Dual-task, Water-based exercise, Cognitive function, Physical fitness, Mental health, Sleep quality, Quality of life

## Abstract

**Background:**

The aquatic environment has characteristics that favor adherence to exercise programs among older adults, and studies have shown that programs in this setting are effective in enhancing various aspects of physical fitness, as well as cognitive function, in this population. Research has explored whether incorporating cognitive tasks into an aquatic exercise program could offer additional benefits. Still, it remains uncertain whether this approach leads to greater improvements in cognitive function compared to aquatic exercise alone. We herein report the protocol of the WaterCog Study, which aims to evaluate the effects of an aquatic aerobic exercise program combined with cognitive tasks, compared to a conventional aquatic aerobic exercise program and a control group, on cognitive function and other health-related outcomes in older women.

**Methods:**

This trial is a randomized, single-blinded, three-arm, parallel, superiority trial. A total of 102 older women will be randomized into one of three groups: 1) an aquatic aerobic exercise program combined with cognitive tasks, 2) a conventional aquatic aerobic exercise program, and 3) a control group. Participants in both exercise groups will complete a 12-week exercise program consisting of two non-consecutive sessions per week. The primary outcome is cognitive function, while secondary outcomes include physical function, cardiovascular, and psychosocial parameters. Outcomes will be measured at baseline, post-intervention, and the 12-week follow-up after the end of the intervention period. The analysis plan will employ an intention-to-treat approach and per-protocol criteria.

**Discussion:**

Our conceptual hypothesis is that both aquatic exercise interventions will significantly improve the investigated outcomes compared to the control group. Additionally, we expect that the integration of cognitive tasks will result in additional benefits in cognitive function, cardiovascular outcomes, and psychosocial parameters, with similar gains in physical function compared to conventional aquatic aerobic exercises in post-intervention and follow-up measures. The findings will contribute to the evidence base for interventions targeting cognitive aging and the development of more effective and engaging exercise programs for older adults.

**Trial registration:**

The trial “Aquatic Exercises Combined with Cognitive Tasks for Older Adults (WaterCog)” was prospectively registered at ClinicalTrials.gov (NCT07156708; https://clinicaltrials.gov/study/NCT07156708) in August 2025.

**Supplementary Information:**

The online version contains supplementary material available at 10.1186/s12877-026-07084-8.

## Background

Population aging has intensified worldwide. According to the World Health Organization (WHO), by 2030, one in six people in the world will be aged 60 years or older. At that time, the share of the population aged 60 years and over is expected to increase from 1 billion in 2020 to 1.4 billion. By 2050, the global population of people aged 60 and older is expected to double, reaching 2.1 billion, while the number of individuals aged 80 or older is projected to triple between 2020 and 2050, reaching 426 million [1]. In Brazil, data from the latest Instituto Brasileiro de Geografia e Estatística (IBGE) Census showed that the number of people aged 60 or over grew by 56% between 2010 and 2022 [2]. Although increased life expectancy is an important achievement, the rise in the proportion of older adults leads to higher government spending, particularly on healthcare systems [3].

The aging process generally involves declines in cognitive function, as well as physical, mental, and cardiovascular health, which reduce the independence of older adults. In terms of physical health, reductions in aerobic capacity and muscle strength are evident, negatively affecting functionality and quality of life [4]. Moreover, aging is commonly accompanied by chronic conditions, including mental and cardiovascular disorders, with depressive symptoms and elevated blood pressure being highly prevalent among older adults [1, 5]. Additionally, cognitive aging can negatively affect several domains of cognitive health that are essential for daily functioning, including processing speed, attention, inhibitory control, memory, cognitive flexibility, and semantic fluency [6]. These declines are linked to morphological and functional changes in the brain, particularly in the prefrontal cortex, and are influenced by factors such as cognitive reserve and lifestyle [7]. Although cognitive decline is also a natural process of aging, in some cases it can progress to mild cognitive impairment and even dementia [7]. Emerging evidence suggests that sedentary and unhealthy lifestyles accelerate brain ageing, while regular physical activity, high cardiorespiratory fitness, or a combination of both, can mitigate cognitive impairment and reduce dementia risk [8].

Given this scenario, implementing non-pharmacological strategies that promote healthy aging is essential to address these challenges and mitigate the economic and social impacts of this process [9]. In addition, social isolation and loneliness are public health concerns faced by older adults, as they negatively impact physical and cognitive health [10, 11]. Regular physical exercise plays a key role, as it helps mitigate several deleterious effects of aging and contributes to a healthy aging phenotype [12]. Furthermore, participation in exercise programs increases social interaction, promotes a sense of belonging, and encourages regular engagement in physical activities [13].

In this context, aquatic exercises, typically performed in group settings, have characteristics that favor adherence to exercise programs among older adults. The unique physical properties of water, such as hydrostatic pressure and thermal conductivity, provide benefits in hemodynamic, neuroendocrine, and metabolic parameters [14]. Water immersion has also been shown to increase cerebral blood velocities [15], and aquatic exercise has also exhibited a greater increase in acute cerebral blood flow compared to similar land-based activity in young adults [16, 17]. Furthermore, buoyancy provides a lower impact on lower limb joints [18] and offers a safer environment that minimizes the fear of falling, a widely recognized barrier to exercise participation in this population [19].

It is well established in the literature that aquatic exercise programs are effective in improving multiple components of physical fitness and overall quality of life in older individuals [20–28]. Moreover, aquatic exercise has been shown to promote beneficial effects on blood pressure regulation, contributing to cardiovascular health in older adults [29]. In turn, evidence regarding its influence on sleep quality and mental health outcomes, such as anxiety and depressive symptoms, remains scarce, with only a few studies specifically addressing these aspects among older adults [30, 31]. Recently, its role in cognitive function has also been acknowledged. Aquatic exercise interventions have demonstrated significant improvements in biochemical and physiological biomarkers associated with neuroplasticity and brain health [32, 33]. In addition, significant increases were observed in neuropsychological tests after programs involving different aquatic exercises in older adults [26, 34, 35].

These findings reinforce the potential of aquatic exercise not only in promoting cognitive function but also in exploring interventions that integrate motor and cognitive components. In this scenario, Schaefer et al. [36] evaluated adults under single- and dual-task conditions, combining cognitive and motor tasks, and found significantly fewer errors in the aquatic environment than on land. Regarding chronic adaptations, studies have investigated whether incorporating cognitive tasks into an aquatic exercise program provides additional benefits for older adults. As a result, dual-task aquatic programs have been shown to be effective in improving cognitive function and functional mobility in older adults [37, 38]. In addition, Meekum et al. [39] compared the effects of an aquatic exercise program with and without cognitive training. They found that combining aquatic exercise with cognitive training enhanced cognitive functions more effectively than exercise alone in older adults with mild cognitive impairment. On the other hand, Sato et al. [40], with a similar design but involving participants without cognitive impairment, observed that the group performing aquatic exercise combined with cognitive tasks showed improved cognitive function, whereas the group performing conventional aquatic exercise showed improved physical function. It is noteworthy that the latter studies had methodological limitations regarding the experimental design and exercise programs, which limit the generalizability of the results.

Although the benefits of dual-task aquatic exercise for older adults are promising, it has not yet been proven whether the inclusion of cognitive tasks provides superior gains in cognitive function compared to the same aquatic exercise program alone. On the other hand, the inclusion of cognitive tasks could interfere with gains in physical fitness and associated mechanisms due to limited attentional capacity [41]. Moreover, little is known about how this approach may affect cardiovascular health and psychosocial outcomes, including symptoms of anxiety and depression, sleep quality, and quality of life. Additionally, the latest global consensus on exercise recommendations for older adults emphasizes that multicomponent interventions, which include cognitive tasks, contribute to improving cognition and functional capacity during aging [12]. Thus, interventions involving aquatic exercise programs that provide adaptations in various components of physical fitness, conducted in combination with cognitive tasks, emerge as an innovative strategy, aligned with current recommendations for healthy aging. Therefore, there is a lack of randomized clinical trials with high methodological rigor to clarify these issues, despite the importance of the topic for the health of older adults.

Therefore, we aim to examine the effects of a 12-week aquatic aerobic exercise program combined with cognitive tasks on cognitive function, physical function, cardiovascular, and psychosocial parameters in older women, compared to a conventional aquatic aerobic exercise program and a control group. We hypothesize that both exercise programs will significantly enhance the measured outcomes compared to the control group. Additionally, we expect the aquatic aerobic exercise program with cognitive tasks to have a more pronounced positive effect on cognitive function, as well as on cardiovascular and psychosocial parameters, compared to conventional aquatic aerobic exercise, with similar responses in physical function.

## Methods

### Patient and public involvement

Participants or members of the public were not involved in the design, conduct, reporting, or dissemination plans of this research. The trial design and intervention protocols were developed by the research team based on previous studies conducted with similar older adult populations and on established recommendations for aquatic exercise programs. The acceptability and safety of the exercise sessions will be monitored throughout the intervention period, and participant feedback will be taken into consideration when interpreting the study findings.

### Trial design and setting

This trial is a randomized clinical trial employing a 1:1:1 allocation ratio. It was designed as a superiority trial with three parallel groups, and the outcome assessors and data analysis were blinded. This trial is being conducted at the School of Physical Education and Physiotherapy (ESEF) of the Federal University of Pelotas (UFPel), Brazil. The present study protocol was designed in accordance with the SPIRIT Statement 2025 [42].

### Eligibility criteria

The inclusion and exclusion criteria for participants are defined as follows:

#### Inclusion criteria

Older women aged 60 and 75 years;

Being physically inactive, meaning not participating in structured physical exercise for at least six months (regular exercise is defined as engaging in any physical training for a minimum of 20 min on two or more days a week).

#### Exclusion criteria

History of cardiovascular disease (except controlled hypertension);

Osteoarticular limitations for the practice of exercises;

Being illiterate due to the self-completion questionnaires and the characteristics of the intervention;

Diagnosis of dementia, schizophrenia, or major depressive disorder.

#### Rationale for sex-specific inclusion:

The study includes only older women to account for potential sex-related differences in aging trajectories relevant to the outcomes of interest, thereby reducing sex-related sample heterogeneity. In addition, aquatic exercise programs typically show greater participation among older women.

### Interventions and comparator

Participants will be randomized into one of three groups: an aquatic aerobic exercise program combined with cognitive tasks, a conventional aquatic aerobic exercise program, or a control group. Aquatic exercises will be standardized as exclusively aerobic due to their demonstrated effectiveness in improving physical function within the proposed 12-week period, as reported in previous studies [20, 21, 23–25]. Accordingly, both intervention groups will perform the same aquatic aerobic exercise program, while the group including cognitive tasks will receive this additional component, which is expected to account for potential differences between the groups. Participants allocated to the control group will not engage in any structured physical or cognitive interventions during the 12-week period. This design will allow us to determine whether any observed improvements in the intervention groups can be attributed to the exercise programs and their inherent characteristics, including the potential contribution of attention and social interaction effects associated with supervised group-based activities, rather than to external factors.

#### Aquatic exercise intervention groups

The intervention will take place at the thermal pool of Brilhante Club in Pelotas. The pool water temperature will be maintained between 30 and 32 °C, with participants immersed to a depth level between the xiphoid process and the shoulders. Participants in both exercise groups will undertake a 12-week exercise program, consisting of two weekly sessions on non-consecutive days. Each session will last 45 min, comprising a 5-min warm-up, 35 min of aerobic exercises, and a 5-min cool-down and stretching. The group with cognitive tasks will perform this additional component during the main part of the exercises. Both exercise groups will undergo one familiarization session with aquatic exercises and learn to use the Borg’s 6–20 rating of perceived exertion (RPE) scale before the start of the intervention. The exercise sessions will be conducted in groups of 8 to 12 participants and delivered by a physical education professional with a bachelor’s degree (MBG) and expertise in aquatic exercise prescription, under the supervision of the principal investigator (CLA). A second instructor will provide in-pool assistance throughout the sessions.

##### Conventional aquatic aerobic exercise program

**Fig. 1:**
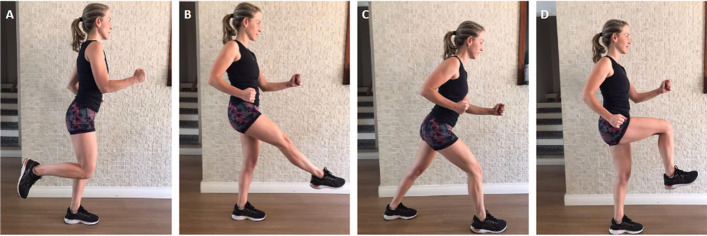
Aquatic exercises included in the sessions: **A** butt kick, **B** frontal kick, **C** cross-country skiing, and **D** stationary running

The aquatic exercise sessions will include the following exercises: butt kick (Fig. 1A), frontal kick (Fig. 1B), cross-country skiing (Fig. 1C), and stationary running (Fig. 1D). These exercises are commonly used in aquatic exercise classes and provide controlled osteoarticular impact, being safe for older women [18].

The program periodization is presented in Table 1. Training intensity will be controlled using the Borg 6–20 RPE scale [43]. The adopted strategy is based on interval training, which alternates periods of high-intensity effort with low-intensity active recovery. Each block will consist of 4 min of effort, with one minute for each exercise (butt kick, frontal kick, cross-country skiing, and stationary running), followed by 1 min of active recovery, also performed using the stationary running exercise. To facilitate monitoring, an RPE scale (0.90 × 1.20 m) will be positioned outside the pool, in front of the participants.

**Table 1:** Periodization of the 12-week aquatic aerobic exercise program

Weeks	Sets	Exercises	Duration	Intensity	Total Duration
1–4	7	Butt Kick	4 min	RPE 13	35 min
		Frontal Kick			
		Cross-Country Skiing			
		Stationary Running			
		Stationary Running	1 min	RPE ≤ 11	
5–8	7	Butt Kick	4 min	RPE 14	35 min
		Frontal Kick			
		Cross-Country Skiing			
		Stationary Running			
		Stationary Running	1 min	RPE ≤ 11	
9–12	7	Butt Kick	4 min	RPE 15	35 min
		Frontal Kick			
		Cross-Country Skiing			
		Stationary Running			
		Stationary Running	1 min	RPE ≤ 11	

*RPE* rating of perceived exertion

##### Aquatic aerobic exercise program combined with cognitive tasks

This group will perform the same aerobic exercise program as the conventional group. Additionally, cognitive tasks will be included during each 1-min active recovery, for a total of 7 min per session. Cognitive training will encompass different domains of cognition in all sessions, including processing speed, attention, inhibitory control, memory, cognitive flexibility, and semantic fluency. The program's targeted cognitive domains were selected for their recognized importance for cognitive health in later life. The selected cognitive tasks are closely related to executive functions, which are essential for everyday functioning. The structure of cognitive training is detailed in Table 2.

**Table 2:** Description of the cognitive tasks used in the program

Domain	Cognitive task	Duration
Processing speed	Listen to the command and execute it as quickly as possible (e.g., touch your head, shoulders, hips, and knees)	1 min
Attention	Count the number of times a previously chosen number is mentioned in a spoken sequence	1 min
Inhibitory control	Perform the opposite command to the statement (e.g., wave your hands only when a specific word is said, and do not react to the others	1 min
Memory	Execute the command previously associated with the color of the plate shown (e.g., red plate – alternating push forward; green plate – elbow flexion and extension)	1 min
Cognitive flexibility	Say a word related to the previous word (e.g., car—tire—rubber—pencil)	1 min
Memory and attention	Repeat the word said previously and add a new one in sequence (e.g., blue; blue and pink; blue, pink, and green…)	1 min
Semantic fluency	In pairs, one-participant names as many words related to a specific topic (e.g., cities, colors, or fruits) as possible in 30 s, while the other counts; afterward, the roles are switched between participants	1 min
Total volume/session		7 min

These domains support daily activities such as quickly evaluating the distance and speed of vehicles when crossing the street (processing speed); maintaining focus while performing domestic tasks without becoming distracted (attention); resisting the urge to stand up abruptly when hearing the phone ring, thereby reducing the risk of falls (inhibitory control); remembering to take medications at the correct times or recalling recently received instructions (memory); adapting to unexpected changes in daily routines, such as modifying a planned route or strategy when circumstances change (cognitive flexibility); and naming objects, foods, or places efficiently during conversations, thereby facilitating communication and reducing pauses or word-finding difficulties (semantic fluency).

The cognitive tasks incorporated into the intervention were designed to increase cognitive load by engaging multiple cognitive domains during simultaneous motor execution, thereby simulating real-world dual task demands. To ensure continuous stimulation and reduce adaptation, task commands varied across sessions, and new commands targeting all cognitive domains were introduced within each mesocycle. Although the intervention did not include a formally structured progression of cognitive load or task complexity across mesocycles, this variability was intended to progressively challenge participants. Furthermore, the cognitive tasks were developed to engage the targeted domains during exercise while avoiding direct overlap with outcome-assessment tasks, thereby minimizing potential learning effects and preserving the validity of the cognitive outcome measures.

#### Control group

The control group will be instructed to maintain their usual physical activity habits and cognitive routines.

#### Criteria for discontinuing or modifying allocated interventions

Participants may discontinue the study at any time if they lose interest or are no longer willing to continue. Participation will be interrupted for individuals allocated to any group in case of safety concerns, such as medical advice or a health event that precludes attendance at intervention sessions.

#### Strategies to improve adherence to interventions

The aquatic exercise intervention groups will receive messages at the beginning of each week, reinforcing the date, time, and location of the sessions. A member of the research team will make phone calls or WhatsApp messages to inquire about adverse events if a participant misses a session in any of the intervention groups. The call schedule will be discontinued for participants who withdraw from the study. Participants assigned to the control group will be informed upon allocation that they will receive the aquatic exercise program after completing their participation in the study.

#### Concomitant care permitted or prohibited during the trial

Participants will be instructed not to engage in any other activities involving exercise or cognitive training during the study period. Participants are also advised to maintain their regular eating habits and continue their treatment for any existing illnesses.

### Outcomes

The primary outcome of the study is executive function (verbal fluency subtype), assessed by the Controlled Oral Word Association Test (COWAT; total F-A-S score), which captures aspects of inhibitory control, cognitive flexibility, and verbal initiation. The primary endpoint is the post-intervention time point. The primary analysis will focus on between-group differences at post-intervention, with additional longitudinal analyses examining changes from baseline and the maintenance of effects at follow-up (12 weeks after the end of the intervention).

The secondary outcomes of the study will be presented as group means and include:

Cognitive function, which will be assessed at baseline, post-intervention, and at the 12-week follow-up after the end of the intervention time points:

- Attention span and short-term memory, measured by the Digit Span Test – Forward (DST–F);
- Processing speed and visual attention, measured by the Trail Making Test – part A (TMT–A);
- Executive function (set-shifting ability), measured by the Trail Making Test – part B (TMT–B);
- Self-perceived cognitive failures, measured by the Cognitive Failures Questionnaire (CFQ).

Physical Function, which will be assessed at baseline and post-intervention time points:

- Aerobic fitness, measured by the 6-min Walk Test (6MWT);
- Lower limb strength, measured by performance in the 30-s Chair Stand Test;
- Upper limb strength, measured by performance in the Arm Curl Test;
- Lower limb flexibility, measured by the Sit-and-Reach Test (Wells Bench);
- Agility and dynamic balance, measured by performance in the Timed Up and Go (TUG) Test;
- Dual-task performance, measured using the TUG combined with a simultaneous cognitive task.

Cardiovascular parameters, which will be assessed at baseline and post-intervention time points:

- Office blood pressure, measured using a calibrated and automated oscillometric device;
- Heart rate variability, measured using a heart rate monitor;
- Blood pressure reactivity, measured using the auscultatory method during the Stroop Test for 3 min;
- Heart rate reactivity, measured using a heart rate monitor during the Stroop Test for 3 min.

Psychosocial parameters, which will be assessed at baseline and post-intervention time points:

- Depressive and anxiety symptoms, measured by the Hospital Anxiety and Depression Scale (HADS);
- Sleep quality, measured by the Pittsburgh Sleep Quality Index (PSQI);
- Quality of life, measured by the World Health Organization Quality of Life—BREF (WHOQOL-BREF) questionnaire.

### Harms

Adverse events will be systematically collected at the end of every week and classified based on their severity (i.e., mild, moderate, or severe), predictability (i.e., expected or unexpected), and potential relationship to study procedures (i.e., definitely related, possibly related, or unrelated). All adverse events are reviewed by a multidisciplinary team that includes at least two clinical experts (AOS, JGR, TR, FBO).

### Participant timeline

The trial enrollment, interventions, and assessments schedule is presented in Table 3.

**Table 3:** Time scheme for the study conduction

	Study period								
	Enrollment	Baseline measures		Allocation	Post-allocation		Close out		
	T −1	T0^a^		T1	T2	T3^b^	T4^a^		T5
Time point description		Evaluation visit 1	Evaluation visit 2		Intervention start	Intervention End	Final evaluation visit 1	Final evaluation visit 2	Follow-up
Enrollment									
Eligibility screening	X								
Informed consent	X								
Allocation				X					
Interventions									
Aquatic aerobic exercise program combined with cognitive tasks					X	X			
Conventional aquatic aerobic exercise program					X	X			
Control group					X	X			
Assessments									
*Primary outcome*									
Objective cognitive function by COWAT			X					X	X
*Secondary outcomes*									
Objective cognitive function by DST–F			X					X	X
Objective cognitive function by TMT–A/B			X					X	X
Self-perceived cognitive function		X					X		X
Functional tests			X					X	
Heart rate variability			X					X	
Office blood pressure		X	X				X	X	
Blood pressure and heart rate reactivity			X					X	
Depressive and anxiety symptoms		X					X		
Sleep quality		X					X		
Quality of life		X					X		
*Other outcomes*									
Cognitive status		X					X		
Sociodemographic and clinical characteristics		X					X		
Anthropometric measurements			X					X	
Physical activity levels		X					X		
Eating habits		X					X		
Monitoring the intensity of training sessions					X	X			
Adherence assessments					X	X			
Satisfaction questionnaire								X	

*COWAT* Controlled Oral Word Association Test, *DST–F* Digit Span Test – Forward, *TMT* Trail Making Test

^a^period will be no longer than 2 weeks

^b^time between t3 and t4 will be no longer than 2 weeks

### Sample size

The sample size was calculated using G*Power 3.1 software, based on the study’s primary outcome, assessed by the COWAT, using unpublished data from our laboratory. An effect size of ƒ = 0.180 was used, with 90% power and an alpha of 5%. The calculation indicated a required sample of 84 participants, with an additional 20% added to account for potential dropouts, resulting in 102 participants to be recruited.

### Recruitment

The recruitment period began in September 2025 and is expected to be concluded by August 2027. Recruitment strategies will include advertisements in local and regional newspapers, as well as on social media, with contact information provided for interested individuals. Additionally, the study will be publicized in primary health care units (Unidades Básicas de Saúde – UBS – in Portuguese). An initial telephone contact will be made, during which participants will receive information about the purpose of the study and undergo a preliminary screening to determine their eligibility. Eligible individuals will be invited to visit the Neuromuscular Assessment Laboratory (LabNeuro) of the ESEF/UFPel. During this visit, they will be received by one of the researchers in charge (MBG or HSF) who will provide detailed information about the research procedures, including potential risks and benefits, and will clarify any doubts. Finally, participants who agree to participate will be asked to sign an informed consent form in Portuguese, which will be obtained in person by the same trained researcher, and a copy of the signed form will be provided to each participant for their records.

### Randomization

The randomization sequence will be generated on the website www.random.org, with a 1:1:1 proportion and stratified by age group (60–67 and 68–75 years), and MMSE scores (categorized as with or without cognitive decline) by a researcher not involved with recruitment, data collection, or intervention. The sequence is based on randomly sized blocks that are not disclosed to ensure concealment.

### Allocation concealment mechanism

Upon enrollment in the study, each participant will be assigned an internal identifying number (ID) that will be used for the allocation sequence. A blinded researcher will implement the allocation after the conclusion of the baseline evaluation by accessing the randomization list based on the participants’ IDs. Participants will be allocated into one of the three groups (aquatic aerobic exercise program combined with cognitive tasks, conventional aquatic aerobic exercise program, or control group) and informed about their intervention via telephone or message.

### Implementation

LSA is the researcher responsible for generating the allocation sequence, CLA is the researcher responsible for enrolling participants, and VHGP is the researcher responsible for assigning participants to interventions.

### Blinding

Blinding will be applied to outcome assessors and data analysts responsible for evaluating both primary and secondary outcomes. Due to the nature of the interventions, blinding is not feasible for the staff conducting the exercise sessions or the participants. To maintain assessor blinding, participants will be instructed not to disclose their group allocation or discuss intervention details during outcome assessments. In the case of unintentional unblinding for any reason, researchers involved will notify the steering committee. This information will be documented for internal control purposes.

### Data collection methods

During the first visit, after signing the consent form, participants will undergo an initial office blood pressure assessment. Afterward, they will complete self-reported questionnaires related to cognitive status, eating habits, mental health, self-perceived of cognitive function, physical activity levels, quality of life, and sleep quality.

On the second visit, participants will initially remain seated at rest for 10 min, during which heart rate variability will be measured. Following this resting period, office blood pressure will be assessed, and participants will proceed with the blood pressure and heart rate reactivity protocol using the Stroop test as a mental stressor. In the sequence, they will complete the cognitive assessments. Finally, anthropometric measurements will be taken, and functional tests will be performed.

All outcome assessments will be conducted by trained and experienced assessors using standardized procedures. The same researcher will apply each test at baseline, post-intervention, and follow-up assessments, with all evaluations conducted during the same time of day across time points to ensure consistency. Questionnaires will be administered individually in a private room to ensure participant comfort and confidentiality. The post-intervention assessments will begin 48 h after the last session. To minimize participant burden and optimize feasibility, the assessment protocol was pilot tested to refine test sequencing, rest periods, and overall logistics, ensuring efficient and standardized data collection.

All randomized participants will undergo outcome assessments, regardless of their participation status or completion. Participants who withdraw from the study at any time after randomization will still be invited to complete the final assessments and follow-up, ensuring inclusion of data in the intention-to-treat analysis.

#### Measurement of the primary outcome

##### Objective cognitive function

The COWAT will be used to assess verbal fluency as an aspect of executive function [44]. In this test, participants must say as many words as possible that start with the letters “F,” “A,” and “S” within 1 min for each letter. Proper names, repeated words, and variations in gender, number, and conjugation are not considered. A higher number of words in each test indicates better verbal fluency.

#### Measurements of secondary outcomes

##### Objective cognitive function

The DST-F will be used to assess attention span and short-term memory [45]. The test requires participants to verbally recall a sequence of numbers dictated by the examiner. It begins with three numbers ranging from 0 to 9, read at 1-s intervals, which participants must remember in the correct order. The sequence increases by one digit if pronounced correctly. If participants fail to recall the correct sequence twice in a row, the test ends. The number of digits correctly recalled is recorded as the test result, with a higher score indicating better performance.

The TMT will be used to assess different components of cognitive processing related to attention and executive functioning [46]. In the first part of the instrument (TMT–A), which measures visual attention and processing speed, participants must draw a line connecting the numbers 1 to 25 in ascending order. In the second part (TMT–B), which measures set-shifting ability (i.e., a core component of cognitive flexibility within executive function), participants connect numbers (1–13) and letters (A–L) in an interleaved numerical and alphabetical order. Participants are instructed to maintain pencil-and-paper contact throughout the test, and shorter completion times indicate better performance.

##### Self-perceived cognitive function

The Brazilian version of the Cognitive Failures Questionnaire (CFQ) will be used to measure the frequency of cognitive failures in everyday life, including lapses in attention, memory problems, and failures in executing intentional actions [47]. The questionnaire consists of 25 self-administered items, with responses on a Likert-type scale from 0 to 4, which assess the frequency of cognitive failures in different daily contexts. The total score ranges from 0 to 100 points, with higher scores indicating a greater perceived frequency of cognitive failures and, therefore, poorer self-perception of cognitive function.

##### Aerobic fitness

The 6MWT will be performed to measure aerobic fitness [48]. The course proposed in the original test is a 45.72 m rectangular course. The course will be adapted for a straight line of 30 m in length, demarcated with cones every 3 m. Participants are instructed to walk for 6 min in a flat 30 m course, where the total distance walked “as fast as possible” is assessed.

##### Lower limb strength

The 30-s Chair-Stand test will be performed to measure the strength of the lower limbs [48]. Participants are instructed to sit and stand up from a chair 43 cm high from the seat, without the aid of the upper limbs, as many times as possible for 30 s. The total number of complete repetitions performed within the 30 s is recorded as the result, with higher values ​​indicating greater lower body strength.

##### Upper limb strength

The Arm Curl test will be performed to measure the strength of the upper limbs [48]. Starting at full elbow extension and holding a 2 kg dumbbell in each hand, participants are instructed to perform the maximal number of elbow crunches over the full range of motion for 30 s. The test is performed with both upper limbs. The total number of complete repetitions performed within the 30-s time frame is recorded as the result, with higher values indicating greater upper body strength.

##### Lower limb flexibility

The Sit-and-Reach test will be performed using a Wells Bench to assess lower limb flexibility, particularly the hamstring and lower back muscles [49]. Participants sit barefoot on the floor with their legs extended and together, knees fully extended, and the soles of their feet flat against the front of the bench. With arms extended and hands overlapping, they slowly bend forward from the hips, sliding their hands along the measurement scale on the bench, without flexing the knees or using compensatory movements. The maximum reach is held for approximately 2 s, and the greatest distance reached in centimeters in three attempts is recorded.

##### Agility and dynamic balance

The TUG test will be performed to measure agility and dynamic balance. Participants are instructed to stand up from the chair (43 cm), turn around a marker that will be 3 m away, and return to the starting position. The shortest time of the two attempts is considered the result.

##### Dual-task performance

Dual-task performance involves simultaneously performing the TUG test and a cognitive task. Participants will be instructed to complete the TUG test while reciting alternating letters (e.g., A, C, E, etc.) [37]. The time taken for the TUG and the number of correctly recited letters are recorded as test results.

##### Blood pressure outcomes

Office mean, systolic, and diastolic blood pressure assessments will be obtained using a calibrated and automated oscillometric device (HEM-7156t, OMRON, China). Participants will be kept in a calm environment for 5 min, after which measurements will be taken in both arms of the participant. Three measurements, with an interval of 1 to 2 min, will be taken in the arm with the highest initial value. The average of the three measurements is considered the subject’s office blood pressure.

##### Blood pressure and heart rate reactivity

Blood pressure and heart rate reactivity will be measured as the difference between values recorded during a mental stressor and those obtained at rest. The Stroop Test [50] will be used to induce mental stress, presented via a video on a screen in front of the participant for 3 min. Every 2 s, a visual stimulus consisting of a word written in a specific color will appear, with the color of the word and its meaning being incongruent (for example, the word "blue" written in red). The participant must respond verbally and as quickly as possible to the color of the letters, ignoring the meaning of the word.

Throughout the test, systolic and diastolic blood pressure will be assessed by the auscultatory method, using a stethoscope and a calibrated aneroid sphygmomanometer (Littmann Classic III, United States), with one reading every minute, for a total of three readings. The reference arm is the same one used for resting BP assessment. Heart rate will be measured at the same time points using a heart rate monitor (H10, Polar, Kempele, Finland). After completing the test, participants will be asked to rate their perceived stress level using a subjective stress scale proposed by Callister et al. [51], with the following ratings: 0 = not at all stressful; 1 = slightly stressful; 3 = very stressful; or 4 = extremely stressful. Additionally, the number of errors made during the test is recorded as an indicator of cognitive performance.

##### Heart rate variability

Heart rate variability (HRV) will be measured with participants seated at rest for 10 min, during which time their heart rate will be continuously recorded using a heart rate monitor (H10, Polar, Kempele, Finland), a validated and widely used device for HRV analysis. Data collection will take place in a quiet environment with a controlled temperature between 24 and 26 °C. Data will be collected via Bluetooth and stored in the Polar Flow app, later exported in a compatible format (.txt or.csv). The final 5 min of the resting period will be considered for analysis, as they offer greater physiological stability.

Data will be processed in Kubios HRV software, utilizing an automatic artifact correction feature with a low threshold filter. HRV analysis will follow the recommendations of the Task Force of the European Society of Cardiology and the North American Society of Pacing and Electrophysiology [52]. Considering the time domain, the parameters Standard Deviation of NN Intervals (SDNN), which reflects the global variability of autonomic activity, and Root Mean Square of Successive Differences (RMSSD), which is an index sensitive to parasympathetic activity and representative of short-term variations in RR intervals, will be determined. In the frequency domain, the Low Frequency (LF, 0.04–0.15 Hz), related to sympathetic-parasympathetic modulation, the High Frequency (HF, 0.15–0.40 Hz), linked to vagal (parasympathetic) modulation, and the LF/HF ratio, an estimate of the autonomic balance between the sympathetic and parasympathetic systems, will be determined. This set of parameters will allow the assessment of cardiac autonomic modulation and potential adaptations induced by the intervention.

##### Depressive and anxiety symptoms

The Brazilian Portuguese adaptation of HADS will be used to assess depressive and anxious symptoms [53]. It is an instrument composed of 14 items, with seven items forming the anxiety subscale and the other seven forming the depression subscale, allowing for the assessment of symptoms from the previous week. Each HADS item has four response options ranging from 0 to 3, and reaches a maximum of 21 points in each subscale. Participants will be instructed to answer the questions based on the last 7 days. Higher scores indicate more severe symptoms and a worse perceived emotional state.

##### Sleep quality

The validated Brazilian Portuguese version of the PSQI will be used to assess subjective sleep quality and related disorders [54]. The questionnaire consists of 19 items, grouped into seven components: subjective sleep quality, sleep latency, sleep duration, habitual sleep efficiency, sleep disorders, use of sleeping medications, and daytime dysfunction. Each component is scored from 0 (no difficulty) to 3 (severe difficulty), and the sum of the scores results in an overall score ranging from 0 to 21; scores greater than 5 indicate poor sleep quality.

##### Quality of life

Quality of life will be assessed using the WHOQOL-BREF questionnaire, developed by the World Health Organization and previously validated in the Brazilian population [55]. The WHOQOL-BREF comprises 26 items divided into four domains: physical, psychological, social relationships, and environment. This instrument is self-administered, utilizing a 5-point Likert scale, and yields standardized scores ranging from 0 to 100, with higher values indicating a better perceived quality of life.

#### Other outcomes

##### Cognitive status

The Brazilian Portuguese version of the MMSE, standardized by Brucki et al. [56], will be used to screen for cognitive decline. In the present study, it will be used to stratify randomization. The questionnaire is divided into two sections: the first, with a maximum score of 21, requires vocal responses regarding temporal and spatial orientation, memory, and attention; the second, with a maximum score of 9, assesses the ability to name, follow commands, write, and copy a polygon. The maximum total score is 30 points, and the test is not timed. Participants’ cognitive function is classified based on cutoff points adjusted for education, as proposed by Brucki et al. [56]. The score is formed by the sum of the items answered correctly; the higher the score, the better the cognitive function.

##### Sociodemographic and clinical characteristics

Sociodemographic data, health history, medical conditions, and medication use will be collected using a standardized questionnaire before the intervention.

##### Anthropometric assessment

Anthropometric outcomes will be measured at baseline and at the post-intervention period. Body mass and height measurements are performed using a digital scale with a stadiometer (Welmy, Santa Bárbara d’Oeste, Sao Paulo, Brazil). The calculation of the body mass index (BMI) is performed using the equation: BMI = body mass (kg)/height^2^ (m). Additionally, waist and hip circumferences will be measured using a measuring tape placed around the navel height and the width of the participants’ hips, respectively, to calculate the waist-hip ratio.

##### Physical activity levels

The self-reported physical activity level will be measured at baseline and post-intervention using the Godin-Shephard Leisure-Time Physical Activity Questionnaire. The Brazilian Portuguese culturally adapted and reliable version of the Godin-Shephard questionnaire will be used [57]. Participants should report the number of times they engage in vigorous, moderate, and light physical activities for more than 15 min per week. The weekly frequencies of intense, moderate, and light activities should be multiplied by nine, five, and three, respectively. The total weekly leisure activity is calculated in arbitrary units from the sum of the products of each component, allowing for to categorization of the individual as insufficiently active or active.

##### Eating habits

The Food Frequency Questionnaire (FFQ) will be used to monitor eating habits. According to the Brazilian Food Guidelines, foods are divided into two groups: in natura/minimally processed foods and processed or ultra-processed foods. Scores are assigned to these groups based on the frequency with which participants report eating them. The instrument includes a list of 16 foods, and scores are assigned to each based on how often participants report consuming them. The scores can range from 0 to 32 points, with higher scores indicating healthier eating habits. An overall healthy eating score is also calculated by summing the points for all evaluated foods, ranging from 0 to 64 points, where higher scores indicate better eating habits. The instrument is applied at baseline and post-intervention.

##### Monitoring the intensity of training sessions

The session rating perceived exertion (sRPE) will be recorded at the end of the last session of each mesocycle using Borg's Category-Ratio 10 scale [58]. The scale in Brazilian Portuguese ranges from 0 to 10, based on perceived effort levels, with 0 indicating no effort (rest) and 10 indicating maximum effort. The sRPE will be collected 5 min after the end of the session, as Christen et al. [59] reported no significant differences between values obtained 5 and 30 min post-exercise.

##### Satisfaction questionnaire

Each participant will answer 7 questions regarding their perception of the intervention using a 5-point Likert scale, in which “1” means “no total agreement” and “5” means “total agreement”. The questionnaire is administered after the intervention to assess participants' perceptions of study satisfaction and safety, as well as benefits for daily life and lifestyle changes.

##### Adherence assessments

Attendance will be monitored through the session’s frequency recording and expressed as the percentage of intervention sessions experienced by a participant, given the total number of scheduled sessions.

#### Plans to promote participant retention and complete follow-up

To improve participant retention and ensure complete follow-up, consistent and clear communication will be maintained with participants to keep their engagement, offer online encouragement, and provide ongoing support to reduce dropouts. Additionally, detailed records of any discontinuation or deviation from intervention protocols, including data collected from participants in these situations, will be kept, ensuring a thorough and transparent analysis of study results.

### Data management

Data are collected on standardized paper forms or through a digital Google Form using a tablet. The method (paper or digital) will be selected according to the characteristics of the questionnaire or test administered, and all data collection will be conducted in person. At the end of every testing day, a researcher will verify missing or inaccurate data and the backup data. Subsequently, double data entry will be conducted for primary, secondary, and additional outcomes.

### Statistical methods

Descriptive data will be presented as means with standard deviations and/or 95% confidence intervals, or as absolute or relative frequencies, when applicable. Generalized Estimating Equations (GEE) will be used to compare primary and secondary outcomes, assuming an unstructured working correlation matrix. In all GEE models, group and time will be treated as categorical variables, and group × time interactions will be included to test intervention effects. Bonferroni-adjusted post hoc tests will be applied when multiple comparisons are performed. The efficacy of the trial will be assessed using a single pre-specified primary outcome and a single primary comparison, consistent with SPIRIT recommendations for clinical trial protocols. Therefore, no multiplicity adjustment across outcomes will be applied. Analyses of secondary outcomes will be considered exploratory, and multiplicity adjustments will be applied only for multiple comparisons within the same outcome (e.g., Bonferroni-adjusted post hoc tests).

Both per-protocol (PP) and intention-to-treat (ITT) analyses will be conducted. In the analysis following the ITT principle, all randomized participants are analyzed in the group to which they were initially assigned, regardless of adherence, treatment receipt, or protocol deviations. Multiple imputations will be used to impute missing data using a specific function on SPSS software. Additionally, PP analyses will be performed to evaluate the robustness of the findings and the effect of adherence to the intervention. Therefore, participants must have at least 70% attendance during the 12-week intervention to be included in the PP analysis. This threshold was defined to ensure sufficient exposure to the training stimulus to plausibly induce adaptations, while accounting for the variability in attendance commonly observed in exercise interventions involving older adults. This cutoff has been adopted in previous aquatic exercise intervention studies conducted by our research group involving older populations under similar conditions [22].

No additional analyses, such as subgroup or sensitivity analyses, are planned for this trial. All statistical procedures will be performed in the SPSS vs. 28.0 program, and the significance level adopted is α = 0.05. Additionally, between-group effect sizes will be calculated using Cohen's d based on change scores and classified as small (≥ 0.2), moderate (≥ 0.5), or large (≥ 0.8) [60, 61].

### Data monitoring committee

The monitoring committee is composed of collaborating researchers (TR, AOS, JGR) who are clinical experts in exercise interventions and biostatisticians not involved in the trial, who will oversee participant safety and trial integrity. The monitoring committee will review adverse events, data quality, and trial conduct at predefined intervals.

### Interim analyses

No interim analyses are planned. There are no expected issues with the intervention that would harm participants or necessitate pausing the trial beforehand.

### Trial monitoring

Trial monitoring is performed by recording attendance at each session, monitoring adverse events at the end of each week, and assessing training intensity at the end of each mesocycle.

## Ethics

### Confidentiality

Each participant’s ID will be used to identify the research data collection instruments. Furthermore, data collected during the research will remain strictly confidential and will only be accessible to the study coordination team. If requested by the research coordination team, anonymous study data may be shared with other researchers. All data produced by this study will be maintained under the responsibility of the lead researcher for a minimum of 5 years.

### Provisions for post-trial care

Upon completing the study, all participants will receive a guide with information on general healthcare and physical activity. Participants in the control group will be invited to participate in an aquatic exercise program in the same format as the training groups. Participants will also receive details about the individual assessments that have been conducted.

## Open science

### Protocol and statistical analysis plan

The whole trial protocol, including the statistical analysis plan, is presented in this article.

### Data sharing

De-identified participant data (including data dictionary), statistical code, and any other materials will be available from the corresponding author upon reasonable request.

### Dissemination plans

Individual results will be delivered to each participant via a report at the end of their participation in the study, including their measurements and interpretations in language adapted for lay public understanding. The results of the complete study will be disclosed to all participants through a lecture at a meeting at the end of the last wave of the study. In addition, they will be sent to the primary health care units collaborating on the project.

Opinion articles, interviews, and reports will be prepared in collaboration with local newspapers to publicize and broadcast the advances and impacts of the project. In addition, content accessible to the general public will be created, including infographics and educational videos, to be posted on social media, with a focus on raising awareness about the impact of physical exercise on the cognitive and physical function of older adults. These will be posted on Labneuro's Instagram (@labneuroufpel), a social media profile aiming at scientific dissemination, which was created in 2020 to publicize our projects and research to the community, reaching both specialist and non-specialist audiences.

Additionally, workshops will be held for healthcare professionals and students of Physical Education, Physiotherapy, and Occupational Therapy courses, as well as other interested parties, to train them in working with dual-task programs and to present the advances achieved through this work.

## Discussion

The WaterCog Study focuses its efforts on understanding the effects of aquatic exercises combined with cognitive tasks, thereby expanding current knowledge about dual-task programs for older women. We are confident in the potential of aquatic exercise to mitigate the deleterious effects of the aging process, given that research on aquatic exercise has shown benefits for physical fitness in older adults since the 1990s [62]. It is essential to note that the latest global consensus on exercise recommendations for older adults emphasizes the benefits of multicomponent interventions, including cognitive tasks, in enhancing cognition and functional capacity with aging [12]. In this context, aquatic exercise programs, enhanced by the properties of water and integrating physical and cognitive stimuli, constitute a safe approach that promotes adherence and complies with current guidelines for promoting healthy aging [12, 14, 18, 63].

Accordingly, this study aims to improve health indicators in older women through group-based exercise programs, targeting cognitive, physical, cardiovascular, and psychosocial outcomes. Both exercise programs are expected to improve these outcomes when compared with the control group. In addition, the aquatic aerobic exercise program combined with cognitive tasks is expected to provide greater benefits, not only in cognitive function but also in cardiovascular and psychosocial outcomes, compared with conventional aquatic aerobic exercise. This assumption is supported by recent studies demonstrating a relationship between cognitive function and cardiovascular as well as psychosocial parameters. Studies have shown that higher blood pressure [64], higher blood pressure reactivity [65], and lower resting heart rate variability [66] are associated with poorer cognitive performance in adults without dementia or stroke. Furthermore, mental health [67], sleep quality [68], and quality of life [69] have also been linked to cognitive function. Exercise interventions that promote better sleep can enhance cognitive performance. Conversely, improved cognitive function can also support better mental health and an overall higher quality of life. These findings emphasize the potential broad benefits of strategies involving dual tasks (i.e., cognitive and motor tasks) in older adults.

On the other hand, gains in physical parameters are expected to be similar in both training groups. Regarding the proposed exercise programs, it is noteworthy that aerobic training programs lasting between 12 and 16 weeks can induce multicomponent adaptations [20–25] due to the multidirectional resistance offered by water. Therefore, the programs were designed to ensure that the physical component was equivalent across both training groups, with the group receiving cognitive tasks being exposed to an additional intervention, allowing any possible differences between the groups to be attributed to this condition. Based on the theory of parallel processing, which states that the central nervous system can process different types of information (e.g., motor and cognitive) simultaneously, but within a limited attentional capacity [41], it is possible that overlapping demands might not only result in similar physical gains but could even lead to reduced physical performance in the dual-task group. To mitigate this potential interference, cognitive tasks were deliberately introduced during active recovery periods, when physical effort levels were lower. This strategy was adopted to reduce competition for attentional resources during phases of higher physical demand, thereby preserving the primary aerobic training stimulus. During active recovery, participants remain physically engaged at a lower intensity, which allows the integration of cognitive challenges without compromising the execution and safety of movements.

The aquatic aerobic exercise program combined with cognitive tasks represents a relatively emerging approach in the aquatic exercise setting, as previous studies have provided limited detail on the structure and dosage of cognitive-motor training in this environment [37–40]. Dual-task approaches combining aerobic exercise and cognitive load have also been described within brain endurance training (BET) frameworks, which typically involve sustained cognitive demands aimed at inducing mental fatigue [70, 71]. Evidence from studies in healthy young adults suggests that continuous dual-task interventions may induce acute cognitive fatigue when sustained for prolonged periods [72, 73]. Although it remains unclear whether similar effects occur in older adults, particularly in the aquatic environment, cognitive fatigue may influence safety, task performance, and adherence, which justifies the dosage proposed in the present study.

Therefore, the present protocol adopted a more conservative, exploratory strategy grounded in established principles of aquatic aerobic training [74]. Exercise sessions were primarily structured to ensure an adequate aerobic training stimulus using an interval-based approach. This strategy was adopted to allow the integration of cognitive tasks during active recovery periods, aiming to minimize potential interference with the planned training volume and intensity. Accordingly, approximately 7 min per session were allocated to cognitive tasks, distributed in seven sets of 1-min cognitive stimuli embedded within these recovery intervals. This structure was considered sufficient to maintain participants’ engagement and interest in the cognitive challenge, while preserving feasibility and safety, and was guided by pilot testing conducted within our research group.

It is important to recognize that the present study may be subject to expectancy bias, particularly regarding self-reported psychosocial outcomes. As with other behavioral exercise interventions, participants’ awareness of participating in an intervention (or being allocated to a control condition) may influence their perceptions and responses to questionnaires. On the other hand, validated instruments, assessor blinding, and standardized assessment procedures were adopted to reduce the potential impact of this bias. Moreover, the use of a passive control group may influence the interpretation of psychosocial outcomes, as attention and social interaction are inherent characteristics of group-based aquatic exercise interventions. Therefore, psychosocial effects should be interpreted within the context of the collective and supervised nature of the intervention. Finally, the inclusion of only older women may limit the generalizability of the findings to older men, and results should therefore be interpreted within the context of this specific population.

In summary, this study aims to enhance health indicators in older women through group exercise programs, focusing on improving both cognitive and physical function. By detailing the scientific rationale behind the WaterCog study, its methodology, and planned analyses, this protocol ensures rigor and transparency, thereby facilitating research reproducibility. The results of this randomized clinical trial are expected to contribute to the advancement of knowledge on safe and effective non-pharmacological interventions for healthy aging, providing valuable insights for clinical practice and the development of health policies targeting older adults.

## Supplementary Information

Supplementary Material 1.

## Data Availability

No datasets were generated or analysed during the current study.

## Abbreviations

6MWT: 6-min Walk Test
BMI: Body mass index
CFQ: Cognitive Failures Questionnaire
CNPq: Conselho Nacional de Desenvolvimento Científico e Tecnológico
COWAT: Controlled Oral Word Association Test
DST-F: Digit Span Test Forward
ESEF: School of Physical Education and Physiotherapy
FFQ: Food Frequency Questionnaire
GEE: Generalized Estimating Equations
HADS: Hospital Anxiety and Depression Scale
HF: High Frequency
IBGE: Instituto Brasileiro de Geografia e Estatística
ID: Identifying number
ITT: Intention-to-treat
LabNeuro: Neuromuscular Assessment Laboratory
LF: Low Frequency
MMSE: Mini Mental State Examination
PP: Per-protocol
PSQI: Pittsburgh Sleep Quality Index
RPE: Rating of perceived exertion
RMSSD: Root Mean Square of Successive Differences
SDNN: Standard Deviation of NN Intervals
TMT: Trail Making Test
TUG: Timed Up and Go
UBS: Unidades Básicas de Saúde
UFPel: Federal University of Pelotas
WHO: World Health Organization
WHOQOL-BREF: World Health Organization Quality of Life – BREF
